# Supplementary material for: BioSeq-Diabolo: Biological sequence similarity analysis using Diabolo
Source: PLoS Comput Biol. 2023 Jun 20;19(6):e1011214. doi: 10.1371/journal.pcbi.1011214 (PMC10313010; doi:10.1371/journal.pcbi.1011214)
Supplement: S1 Table — (DOCX) [file pcbi.1011214.s001.docx]

**S1 Table**. Distribution methods and their descriptions.

| Distribution methods | Descriptions |
| --- | --- |
| SVM | Support Vector Machines [1] |
| RF | Random Forests [2] |
| ERT | Extremely randomized trees [3] |
| KNN | K-nearest neighbours [4] |
| MNB | Naive Bayes classifier for multinomial models [5] |
| GBDT | Traditional Gradient Boosting Decision Tree [6] |
| GOSS | Gradient-based One-Side Sampling [7] |
| DART | Dropouts meet Multiple Additive Regression Trees [8] |
| MLP | Multi-layer Perceptron [9] |

**REFERENCES**

1. Chang C-C, Lin C-J. LIBSVM: A library for support vector machines. ACM Trans Intell Syst Technol. 2011;2(3):27. doi: 10.1145/1961189.1961199.

2. Breiman L. Random Forests. Machine Learning. 2001;45(1):5-32. doi: 10.1023/A:1010933404324.

3. Geurts P, Ernst D, Wehenkel L. Extremely randomized trees. Machine Learning. 2006;63(1):3-42. doi: 10.1007/s10994-006-6226-1.

4. Altman NS. An Introduction to Kernel and Nearest-Neighbor Nonparametric Regression. The American Statistician. 1992;46(3):175-85. doi: 10.1080/00031305.1992.10475879.

5. Watson J. Introduction to Information Behaviour. Internet Reference Services Quarterly. 2016;21(1-2):51-2. doi: 10.1080/10875301.2016.1163634.

6. Jerome HF. Greedy function approximation: A gradient boosting machine. The Annals of Statistics. 2001;29(5):1189-232. doi: 10.1214/aos/1013203451.

7. Ke G, Meng Q, Finley T, Wang T, Chen W, Ma W, et al. Lightgbm: A highly efficient gradient boosting decision tree. Advances in neural information processing systems. 2017;30.

8. Rashmi Korlakai V, Ran G-B. DART: Dropouts meet Multiple Additive Regression Trees. International Conference on Artificial Intelligence and Statistics; 02/212015. p. 489-97.

9. Xavier G, Yoshua B. Understanding the difficulty of training deep feedforward neural networks. International Conference on Artificial Intelligence and Statistics; 2010/03/312010. p. 249-56.
