# Supplementary material for: BioSeq-Diabolo: Biological sequence similarity analysis using Diabolo
Source: PLoS Comput Biol. 2023 Jun 20;19(6):e1011214. doi: 10.1371/journal.pcbi.1011214 (PMC10313010; doi:10.1371/journal.pcbi.1011214)
Supplement: S2 Table — (DOCX) [file pcbi.1011214.s002.docx]

**S2 Table**. Representation methods and their descriptions.

| Representation methods | Descriptions |
| --- | --- |
| ARC-I | Architecture-I for matching two sentences in [1] |
| CDSSM | Learning Semantic Representations Using Convolutional  Neural Networks for Web Search in [2] |
| DSSM | Deep Structured Semantic Models for web search in [3] |
| MV-LSTM | A Deep Architecture for Semantic Matching with Multiple Positional Sentence Representations in [4] |

**REFERENCES**

1. Hu B, Lu Z, Li H, Chen Q. Convolutional neural network architectures for matching natural language sentences. Advances in neural information processing systems. 2014;27.

2. Shen Y, He X, Gao J, Deng L, Mesnil G. Learning semantic representations using convolutional neural networks for web search. Proceedings of the 23rd International Conference on World Wide Web2014. p. 373–4.

3. Huang P-S, He X, Gao J, Deng L, Acero A, Heck L, editors. Learning deep structured semantic models for web search using clickthrough data. Proceedings of the 22nd ACM international conference on Information & Knowledge Management; 2013.

4. Wan S, Lan Y, Guo J, Xu J, Pang L, Cheng X, editors. A deep architecture for semantic matching with multiple positional sentence representations. Proceedings of the AAAI Conference on Artificial Intelligence; 2016.
