# Supplementary material for: BioSeq-Diabolo: Biological sequence similarity analysis using Diabolo
Source: PLoS Comput Biol. 2023 Jun 20;19(6):e1011214. doi: 10.1371/journal.pcbi.1011214 (PMC10313010; doi:10.1371/journal.pcbi.1011214)
Supplement: S3 Table — (DOCX) [file pcbi.1011214.s003.docx]

**S3 Table**. Interaction methods and their descriptions.

| Interaction methods | Descriptions |
| --- | --- |
| aNMM | Ranking Short Answer Texts with Attention-Based  Neural Matching Model  in [1] |
| ARC-II | Architecture-II of convolutional matching model in [2] |
| BiMPM | Bilateral Multi-Perspective Matching for Natural Language Sentences in [3] |
| ConvKNRM | Convolutional Neural Networks for So-Matching N-Grams in  Ad-hoc Search in [4] |
| DIIN | Densely Interactive Inference Network in [5] |
| DRMM | A Deep Relevance Matching Model in [6] |
| DRMMTKS | A Deep Top-K Relevance Matching Model in [7] |
| DUET | Deep neural networks focused on matching using  local and distributed representations of text in [8] |
| ESIM | Enhanced Sequential Inference Model in [9] |
| HBMP | Sentence Embeddings in NLI with Iterative Refinement Encoders in [10] |
| KNRM | A kernel based neural ranking model in [11] |
| MatchLSTM | Match-LSTM and Answer Pointer for machine comprehension in [12] |
| MatchPyramid | A deep architecture for text matching in [13] |
| Match-SRNN | Modeling the Recursive Matching Structure with Spatial RNN in [14] |

**REFERENCES**

1. Yang L, Ai Q, Guo J, Croft WB. aNMM: Ranking Short Answer Texts with Attention-Based Neural Matching Model. Proceedings of the 25th ACM International on Conference on Information and Knowledge Management2016. p. 287–96.

2. Hu B, Lu Z, Li H, Chen Q. Convolutional neural network architectures for matching natural language sentences. Advances in neural information processing systems. 2014;27.

3. Wang Z, Hamza W, Florian R. Bilateral Multi-Perspective Matching for Natural Language Sentences. ArXiv. 2017;abs/1702.03814.

4. Dai Z, Xiong C, Callan J, Liu Z. Convolutional Neural Networks for Soft-Matching N-Grams in Ad-hoc Search. Proceedings of the Eleventh ACM International Conference on Web Search and Data Mining2018. p. 126–34.

5. Gong Y, Luo H, Zhang J. Natural Language Inference over Interaction Space. ArXiv. 2018;abs/1709.04348.

6. Guo J, Fan Y, Ai Q, Croft WB. A Deep Relevance Matching Model for Ad-hoc Retrieval. Proceedings of the 25th ACM International on Conference on Information and Knowledge Management2016. p. 55–64.

7. Yang Z, Lan Q, Guo J, Fan Y, Zhu X, Lan Y, et al., editors. A Deep Top-K Relevance Matching Model for Ad-hoc Retrieval. Information Retrieval; 2018.

8. Mitra B, Diaz F, Craswell N. Learning to Match using Local and Distributed Representations of Text for Web Search. Proceedings of the 26th International Conference on World Wide Web2017. p. 1291–9.

9. Chen Q, Zhu X-D, Ling Z, Wei S, Jiang H, Inkpen D, editors. Enhanced LSTM for Natural Language Inference. In Proceedings of the 55th Annual Meeting of the Association for Computational Linguistics; 2017.

10. Talman A, Yli-Jyrä A, Tiedemann JJNLE. Sentence embeddings in NLI with iterative refinement encoders. 2019;25(4):467-82.

11. Xiong C, Dai Z, Callan J, Liu Z, Power R. End-to-End Neural Ad-hoc Ranking with Kernel Pooling. Proceedings of the 40th International ACM SIGIR Conference on Research and Development in Information Retrieval2017. p. 55–64.

12. Wang S, Jiang J. Machine Comprehension Using Match-LSTM and Answer Pointer. ArXiv. 2017;abs/1608.07905.

13. Pang L, Lan Y, Guo J, Xu J, Wan S, Cheng X. Text Matching as Image Recognition. ArXiv. 2016;abs/1602.06359.

14. Wan S, Lan Y, Xu J, Guo J, Pang L, Cheng X. Match-SRNN: Modeling the Recursive Matching Structure with Spatial RNN. ArXiv. 2016;abs/1604.04378.
