# Supplementary material for: BioSeq-Diabolo: Biological sequence similarity analysis using Diabolo
Source: PLoS Comput Biol. 2023 Jun 20;19(6):e1011214. doi: 10.1371/journal.pcbi.1011214 (PMC10313010; doi:10.1371/journal.pcbi.1011214)
Supplement: S5 Table — (DOCX) [file pcbi.1011214.s005.docx]

**S5 Table**. The performance of BioSeq-Diabolo compared with competing methods in Biological Process Ontology (BPO) for protein function annotation.

| Methods | AUPR^c^ | Fmax^c^ | Smin^c^ |
| --- | --- | --- | --- |
| Naive^1^ | 0.173 | 0.253 | 25.183 |
| DIAMONDScore^1^ | 0.304 | 0.382 | 22.577 |
| DeepGO^1^ | 0.213 | 0.362 | 23.251 |
| DeepGOCNN^1^ | 0.213 | 0.388 | 23.234 |
| BioSeq-Diabolo^b^ | 0.210 | 0.377 | 23.461 |

^a^ The results of the competing methods were obtained from [1]. These competing methods and BioSeq-Diabolo were evaluated on the same test dataset. Therefore, these results can be directly compared;

^b^ The result of the best predictor constructed by BioSeq-Diabolo (integrating top 5 best predictor by using Learning to Rank). The input protein sequence embeddings are extracted by BioSeq-BLM [2] with Position-Specific method [3]. The input GO term embeddings are represented by label embedding matrix reported in [1];

^c^ The performance evaluation indicators were described in **S2 Text** and details of the reported experiments were described in **S3 Text** and **S4 Tex**t.

**REFERENCES**

1. Cao Y, Shen Y. TALE: Transformer-based protein function Annotation with joint sequence–Label Embedding. Bioinformatics. 2021;37(18):2825-33. doi: 10.1093/bioinformatics/btab198.

2. Li H-L, Pang Y-H, Liu B. BioSeq-BLM: a platform for analyzing DNA, RNA and protein sequences based on biological language models. Nucleic Acids Research. 2021;49(22):e129-e. doi: 10.1093/nar/gkab829.

3. Doench JG, Fusi N, Sullender M, Hegde M, Vaimberg EW, Donovan KF, et al. Optimized sgRNA design to maximize activity and minimize off-target effects of CRISPR-Cas9. Nature Biotechnology. 2016;34(2):184-91. doi: 10.1038/nbt.3437.
