# Supplementary figures and images for: BioSeq-Diabolo: Biological sequence similarity analysis using Diabolo
Source: PLoS Comput Biol. 2023 Jun 20;19(6):e1011214. doi: 10.1371/journal.pcbi.1011214 (PMC10313010; doi:10.1371/journal.pcbi.1011214)

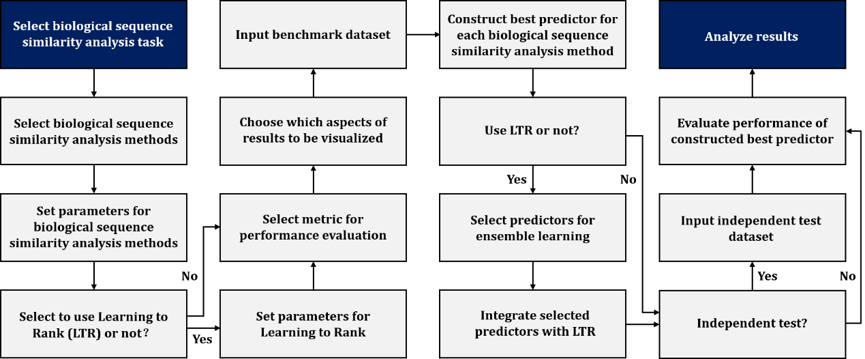

Supplement: S1 Fig — The web server and stand-alone package of BioSeq-Diabolo were developed based on this procedure. (TIF) [file pcbi.1011214.s006.tif]

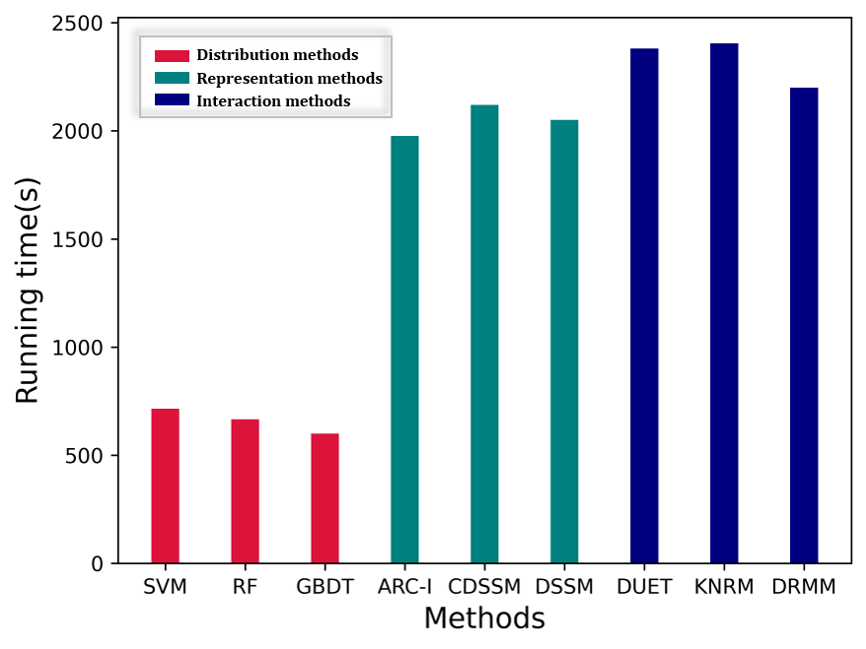

Supplement: S2 Fig — The running time contained training stage and test stage (50000 training examples and 50000 test samples). These experiments were performed on Intel(R) Xeon(R) CPU E5-2660 v3 (2.60 GHz with 10 cores) and memory of 64 G. (TIF) [file pcbi.1011214.s007.tif]
