# Supplementary material for: BioSeq-Diabolo: Biological sequence similarity analysis using Diabolo
Source: PLoS Comput Biol. 2023 Jun 20;19(6):e1011214. doi: 10.1371/journal.pcbi.1011214 (PMC10313010; doi:10.1371/journal.pcbi.1011214)
Supplement: S1 Text — (DOCX) [file pcbi.1011214.s008.docx]

**Learning to Rank**

Learning to Rank (LTR) [1] is widely used in information retrieval and data mining. Based on different loss functions, LTR can be divided into three categories, including pointwise, pairwise and listwise. Among them, listwise achieves the best performance. As an efficient listwise ranking algorithm, LambdaMART combines LamdaRank and MART (Multiple Additive Regression Tree) [2], which is very successful for solving real world ranking problem. In this study, LambdaMART with Normalized Discounted Cumulative Gain (NDCG) [3] loss function was employed. Take circRNA-disease association identification as an example, the loss function $L(\cdot)$ and empirical risk function $R(\cdot)$ of LambdaMART can be formulated as (1) and (2):

$L(F(\boldsymbol{x}^{(i)}), y^{(i)}) = exp(-G_{max, i}^{-1}(k)\sum_{j=1}^{k} \frac{2^{y_{j}^{(i)}}- 1}{{log}_{2}(1+j)}$ (1)

where $k$ denotes the ranking position of disease in predictive disease list, $y_{j}^{(i)}$ is the true label of disease ranked in the $j_{th}$ position for query circRNA $i$, and $G_{max}$ are the normalizing factors calculated by the Ideal Discounted Cumulative Gain (IDCG)

$R(F) = \frac{1}{p}\sum_{i=1}^{p} L(F(\boldsymbol{x}^{(i)}), y^{(i)})$ (2)

where $p$ denotes the number of training circRNAs, and each predictive disease list is ranked according to function F(𝒙). LightGBM [4] is used as the implementation of LTR.

**REFERENCES**

1. Burges C, Shaked T, Renshaw E, Lazier A, Deeds M, Hamilton N, et al., editors. Learning to rank using gradient descent. Proceedings of the 22nd international conference on Machine learning; 2005.

2. Burges CJ. From RankNet to LambdaRank to LambdaMART: An Overview. Learning. 2010.

3. Järvelin K, Kekäläinen J. Cumulated gain-based evaluation of IR techniques. ACM Trans Inf Syst. 2002;20(4):422–46. doi: 10.1145/582415.582418.

4. Ke G, Meng Q, Finley T, Wang T, Chen W, Ma W, et al., editors. LightGBM: A Highly Efficient Gradient Boosting Decision Tree2017: Curran Associates, Inc.
