# Supplementary material for: BioSeq-Diabolo: Biological sequence similarity analysis using Diabolo
Source: PLoS Comput Biol. 2023 Jun 20;19(6):e1011214. doi: 10.1371/journal.pcbi.1011214 (PMC10313010; doi:10.1371/journal.pcbi.1011214)
Supplement: S2 Text — (DOCX) [file pcbi.1011214.s009.docx]

**Performance evaluation indicators**

AUC [1] and AUPR [2] are standard metric in machine learning for evaluating the binary classification performance, especially suitable for highly imbalanced data.

$AUC : Area Under ROC Curve 0 \leq AUC \leq1$ (3)

$AUPR : Area Under Precision-Recall Curve 0\leq AUPR \leq1$ (4)

NDCG (Normalized Discounted Cumulative Gain) [3] is often used to evaluate the ranking quality in search and recommendation tasks, calculated by:

$NDCG = \frac{1}{|\mathbf{Q}|}\sum_{i=1}^{|\mathbf{Q}|} \frac{1}{{IDCG}_{i}}\sum_{j=1}^{d} \frac{2^{y_{j}^{(i)}}- 1}{{log}_{2}(1+j)}$ (5)

where |**Q**| is the number of queries, and ${IDCG}_{i}$ is the ideal discounted cumulative gain for query *i*, while *d* denotes the number of candidate retrieval documents. $y_{j}^{(i)}$ is the true label of the retrieval document ranked in the $j_{th}$ position for query *i*.

ROC [4] is used to evaluate the ranking quality and the ability of retrieval. For example, ROC1 and ROC50 scores are often used for protein remote homology detection. If ROC1 or ROC50 is 1, it indicates the ranking list gets a perfect ranking.

$F_{max}$ and $S_{min}$ are often used in multi-label classification tasks. For example, $F_{max}$ is an official evaluation metric in CAFA [5] for protein function annotation [6]. $F_{max}$ can be calculated by:

$F_{\max} = \max_{t}\left\{ \frac{2\cdot AvgPr(t)\cdot AvgRc(t)}{AvgPr(t) + AvgRc(t)} \right\}$ (6)

where $AvgPr(t)$ and $AvgRc(t)$ are the averaged precision and recall at threshold *t*.

$S_{min}$ computes the semantic distance between real and predicted labels based on information content of the classes [6]. The information content IC(c) [7] is computed based on the annotation probability of the class c:

$IC(c) = -log(Pr(c|P(c))$ (7)

where *P*(*c*) is a set of parent classes of the class *c*. The $S_{min}$ is computed using the following formulas:

$S_{min} = {min}_{t}\sqrt{{ru(t)}^{2} + {mi(t)}^{2}}$ (8)

where $ru(t)$ is the average remaining uncertainty and $mi(t)$ is average misinformation:

$ru(t) = \frac{1}{n}\sum_{i=1}^{n} \sum_{c\in T_{i}-p_{i}(t)} IC(c)$ (9)

$mi(t) = \frac{1}{n}\sum_{i=1}^{n} \sum_{c\in p_{i}(t)-T_{i}} IC(c)$ (10)

**REFERENCES**

1. Bamber D. The area above the ordinal dominance graph and the area below the receiver operating characteristic graph. Journal of Mathematical Psychology. 1975;12(4):387-415. doi: 10.1016/0022-2496(75)90001-2.

2. Fawcett T. An introduction to ROC analysis. Pattern Recognition Letters. 2006;27(8):861-74. doi: 10.1016/j.patrec.2005.10.010.

3. Järvelin K, Kekäläinen J. Cumulated gain-based evaluation of IR techniques. ACM Trans Inf Syst. 2002;20(4):422–46. doi: 10.1145/582415.582418.

4. Gribskov M, Robinson NL. Use of receiver operating characteristic (ROC) analysis to evaluate sequence matching. Computers & Chemistry. 1996;20(1):25-33. doi: 10.1016/S0097-8485(96)80004-0.

5. Zhou N, Jiang Y, Bergquist TR, Lee AJ, Kacsoh BZ, Crocker AW, et al. The CAFA challenge reports improved protein function prediction and new functional annotations for hundreds of genes through experimental screens. Genome Biology. 2019;20(1):244. doi: 10.1186/s13059-019-1835-8.

6. Kulmanov M, Khan MA, Hoehndorf R. DeepGO: predicting protein functions from sequence and interactions using a deep ontology-aware classifier. Bioinformatics. 2018;34(4):660-8. doi: 10.1093/bioinformatics/btx624.

7. Gligorijević V, Renfrew PD, Kosciolek T, Leman JK, Berenberg D, Vatanen T, et al. Structure-based protein function prediction using graph convolutional networks. Nature Communications. 2021;12(1):3168. doi: 10.1038/s41467-021-23303-9.
