# Supplementary material for: BioSeq-Diabolo: Biological sequence similarity analysis using Diabolo
Source: PLoS Comput Biol. 2023 Jun 20;19(6):e1011214. doi: 10.1371/journal.pcbi.1011214 (PMC10313010; doi:10.1371/journal.pcbi.1011214)
Supplement: S3 Text — (DOCX) [file pcbi.1011214.s010.docx]

**Method optimization and Result visualization**

Since there are numerous biological sequence similarity analysis methods in BioSeq-Diabolo, we provide the method optimization function for different tasks. The user can utilize the following command lines for automatically selecting the optimized methods:

| *python sesica_clf.py -data_type homo -bmk_vec bmk_vec.txt -bmk_label pos_label.txt neg_label.txt -clf svm rf ert knn mlp -metric roc@1 -gs_mode 2* |
| --- |

By setting the “-gs_mode” parameter as 2, user can automatically optimize methods based on grid search.

In order to display our analysis results more intuitively, we provide the result visualization function. Users can visualize the results by running the following command line:

| *python sesica_plot.py -data_type homo -clf svm rf ert knn mlp -rank ltr -plot roc prc box polar hp dr dist pie bar -plot_set test* |
| --- |

Then, the users will obtain the visualization results for model performance, similarity network, relevance map, score distribution, contribution of integrated predictors, comparison between embeddings and similarity scores.
