# Supplementary material for: BioSeq-Diabolo: Biological sequence similarity analysis using Diabolo
Source: PLoS Comput Biol. 2023 Jun 20;19(6):e1011214. doi: 10.1371/journal.pcbi.1011214 (PMC10313010; doi:10.1371/journal.pcbi.1011214)
Supplement: S4 Text — (DOCX) [file pcbi.1011214.s011.docx]

**The details of the reported experiments**

1. Details for protein remote homology detection

We train and evaluate on a benchmark dataset constructed based on SCOP1.75 database [1] (<http://bliulab.net/BioSeq-Diabolo/download/>). We split the benchmark into 10 subsets sharing no proteins from the same family, where one subset is test dataset, and the other 9 subsets are used as training dataset. The feature vectors of protein sequences are extracted by 2-Kmer method [2]. The proteins share the same superfamily are defined as positive samples, and the proteins belong to different superfamilies are defined as negative samples. The best predictor is constructed by integrating the top 5 best predictors with Learning to Rank. The construction of the best predictor can be automatically implemented by BioSeq-Diabolo by using the command line on GitHub (<https://github.com/Zimiao1025/Sesica>).

1. Details for circRNA-disease association identification

Our training and independent test dataset are the same as the first application scenario ‘detection of missing associations’ defined in [3]. The input circRNA feature vectors are extracted by BioSeq-Analysis2.0 [4] with PseKNC [5] (parameter 𝜑 is set as 0.5). The input disease feature is represented by semantic similarity score matrix reported in [3]. We concatenate circRNA features and disease features, which are fed into BioSeq-Diabolo for further analysis. The positive circRNA-disease associations are regard as positive samples and the negative circRNA-disease associations are negative samples. BioSeq-Diabolo constructs numerous predictors, and the top-5 predictors are integrated by Learning to Rank. The construction of the best predictor can be automatically implemented by BioSeq-Diabolo by using the command line on GitHub (<https://github.com/Zimiao1025/Sesica>).

1. Details for protein function annotation

Our training and test datasets are constructed based on CAFA3 database [6], and the splits of dataset is from [7]. The ontologies of database include the molecular function ontology (MFO), biological process ontology (BPO) and cellular component ontology (CCO). We compare our method with competing methods in Cellular Component Ontology (CCO) for protein function annotation. The input protein sequence features are extracted by BioSeq-BLM [8] with Position-Specific method [9]. The input GO term embeddings are represented by label embedding matrix reported in [7]. If a sample protein *i* is annotated with function *j*, then the protein-function pair is considered as positive sample. Otherwise, the protein-function pair is considered as a negative sample. The construction of the best predictor can be automatically implemented by BioSeq-Diabolo by using the command line on GitHub (<https://github.com/Zimiao1025/Sesica>).

**REFERENCES**

1. Murzin AG, Brenner SE, Hubbard T, Chothia C. SCOP: a structural classification of proteins database for the investigation of sequences and structures. J Mol Biol. 1995;247(4):536-40. Epub 1995/04/07. doi: 10.1006/jmbi.1995.0159. PubMed PMID: 7723011.

2. Liu B, Liu F, Wang X, Chen J, Fang L, Chou K-C. Pse-in-One: a web server for generating various modes of pseudo components of DNA, RNA, and protein sequences. Nucleic Acids Research. 2015;43(W1):W65-W71. doi: 10.1093/nar/gkv458.

3. Wei H, Xu Y, Liu B. iCircDA-LTR: identification of circRNA–disease associations based on Learning to Rank. Bioinformatics. 2021;37(19):3302-10. doi: 10.1093/bioinformatics/btab334.

4. Liu B, Gao X, Zhang H. BioSeq-Analysis2.0: an updated platform for analyzing DNA, RNA and protein sequences at sequence level and residue level based on machine learning approaches. Nucleic Acids Research. 2019;47(20):e127-e. doi: 10.1093/nar/gkz740.

5. Chen W, Lin H, Chou K-C. Pseudo nucleotide composition or PseKNC: an effective formulation for analyzing genomic sequences. Molecular BioSystems. 2015;11(10):2620-34. doi: 10.1039/C5MB00155B.

6. Zhou N, Jiang Y, Bergquist TR, Lee AJ, Kacsoh BZ, Crocker AW, et al. The CAFA challenge reports improved protein function prediction and new functional annotations for hundreds of genes through experimental screens. Genome Biology. 2019;20(1):244. doi: 10.1186/s13059-019-1835-8.

7. Cao Y, Shen Y. TALE: Transformer-based protein function Annotation with joint sequence–Label Embedding. Bioinformatics. 2021;37(18):2825-33. doi: 10.1093/bioinformatics/btab198.

8. Li H-L, Pang Y-H, Liu B. BioSeq-BLM: a platform for analyzing DNA, RNA and protein sequences based on biological language models. Nucleic Acids Research. 2021;49(22):e129-e. doi: 10.1093/nar/gkab829.

9. Doench JG, Fusi N, Sullender M, Hegde M, Vaimberg EW, Donovan KF, et al. Optimized sgRNA design to maximize activity and minimize off-target effects of CRISPR-Cas9. Nature Biotechnology. 2016;34(2):184-91. doi: 10.1038/nbt.3437.
