# Supplementary material for: BioSeq-Diabolo: Biological sequence similarity analysis using Diabolo
Source: PLoS Comput Biol. 2023 Jun 20;19(6):e1011214. doi: 10.1371/journal.pcbi.1011214 (PMC10313010; doi:10.1371/journal.pcbi.1011214)
Supplement: Attachment — Submitted filename: Reponse letter.docx [file pcbi.1011214.s012.docx]

Dear Prof. Dr. Maxwell Wing Libbrecht,

Thanks for your e-mail and the attached review reports from three reviewers. In this revision, we have carefully revised all the points arising from the reviewers. Our point-to-point responses can be summarized as follows. For clarity, the reviewers’ comments are colored in green beginning with C (comment), while our responses in blue beginning with **R** (response).

**Reviewer #1**

**C.** Li and Liu introduced a new method called BioSeq-Diabolo to calculate the biological sequence similarities. Biological sequence similarity analysis is an important task in bioinformatics, and efficient methods are desired. Generally, BioSeq-Diabolo is interesting, because it uses the techniques derived from the field of natural language processing, bringing new techniques and concepts to solve this task. The corresponding webserver has been constructed, and the standalone package has been released as well. I have tested both the webserver and the package, and they worked well as described. I believe that they will be particularly interesting for the researchers who are working on the related fields. I have the following comments to improve the presentation of this manuscript.

**R.** We very much appreciate Reviewer #1’s overall very positive comments.

**C1.** From the webserver, I understand that there are several pipeline software tools as listed in the right part. However, the relationships between these software tools and BioSeq-Diabolo should be explained in the webserver site as well. As a result, the users are able to use these software tools for their own tasks and aims.

**R1.** Very constructive suggestions. We have added the descriptions of the relationships between the related software tools and BioSeq-Diabolo on the home page of webserver (<http://bliulab.net/BioSeq-Diabolo/#softwares> ).

**C2.** The information or references of the datasets provided in the Download section (http://bliulab.net/BioSeq-Diabolo/download/) should be given.

**R2.** Good suggestion. We have provided more information and references of the datasets in the Download section (<http://bliulab.net/BioSeq-Diabolo/download/>).

**C3.** I have downloaded the stand-alone package from http://bliulab.net/BioSeq-Diabolo/static/download/Sesica.tar.gz, and I felt this package is easy to use, especially for the large dataset analysis aims. However, I failed to find the files for the examples reported in the main text. I suggest Li and Liu providing this information so as to help the users reproducing the reported experiments.

**R3.** Good suggestion. We have provided all datasets reported in the main text in the Download section of web server. After the users download the dataset and source code, they can decompress the dataset file to the "/data" folder of stand-alone package, and then run the experiment according to the corresponding command line in <https://github.com/Zimiao1025/Sesica> or the manual of stand-alone package.

**C4.** The figure 2 is confusing. More information should be provided in its legend.

**R4.** Good point. we have added more detailed description of Figure 2 in its legend.

**C5.** The structure shown in figure 4 is clear, which is very similar as the Diabolo, but the input and output shown in the top part flowchart is not clear. The two important components should be shown in different colors.

**R5.** Good suggestion. We have modified Figure 4.

**C6.** In the current study, Li and Liu used four performance measures to evaluate their performance. Did they follow other studies, and why?

**R6.** Good point. In this version, we add two more evaluation indicators and provide detailed description of the evaluation indicators in the supplementary materials. In order to comprehensively and fairly evaluate the performance of different models in BioSeq- Diabolo, we refer to many previous studies for different tasks in bioinformatics. Detailed information and references are shown in in the Supplementary Materials.

**C7.** For a given methods in BioSeq-Diabolo, it is difficult for the users to select the best one for their own tasks. Did Li and Liu consider this problem? It will be more useful to add the function to automatically select the optimized methods for different tasks. Furthermore, there are several parameters for the different methods. The parameter optimization function should also be added.

**R7.** Very constructive point. Since there are numerous biological sequence similarity analysis methods in BioSeq-Diabolo, we did take the method optimization for different tasks into consideration. The details information is shown in the Supplementary Materials.

**C8.** The result visualization part is elegant and clear, but I cannot find the corresponding figures when using the standalone package. Can Li and Liu also add these visualization function into the standalone package as well?

**R8.** Good point. We have integrated visualization functions into the stand-alone package. Please refer to the Supplementary Materials.

**C9.** This manuscript presented a very useful tool for biological sequence similarity analysis, but more discussions of its potential applications should be given in the conclusion section in order to attract the readers of Plos computational biology.

**R9.** Constructive suggestion. We have added more discussions of BioSeq-Diabolo’s potential applications in section conclusion.

**Reviewer #2**

**C.** The authors established the web server and the stand-alone package for biological sequence similarity analysis called BioSeq-Diabolo. Its performance was well evaluated for different biological sequence similarity analysis problems. BioSeq-Diabolo is easily combined with their previously established software tools, and it is the first comprehensive platform incorporating various methods from natural language processing (NLP). It is reasonable to conduct the biological sequence similarity analysis based on the NLP methods considering the similarities between the biological sequences and the natural language sentences.

**R.** We very much appreciate Reviewer #2’s overall positive comments.

**C1.** The heterogeneous biological sequence similarity analysis shown in BioSeq-Diabolo will contribute to many problems in bioinformatics, such as non-coding RNA and disease association prediction. In the current version of the manuscript, it is not clear how to combine the heterogeneous features (such as non-coding RNAs and diseases) to make the prediction. More explanations are needed.

**R1.** Very good suggestion. While there are many ways to combine the heterogeneous features, in this work, BioSeq-Diabolo supports user-defined features as inputs.

**C2.** The information shown in table 1 clearly indicates that BioSeq-Diabolo and their previous method BLM are complementary, dealing with different problems. For example, Diabolo is for biological sequence similarity analysis, and BLM is for classification or sequence labelling problem in bioinformatics. More explanations of their relationships are need in both the main text and the web site of Diabolo.

**R2.** Good point. The biological sequence features extracted by BioSeq-BLM can be served as the input of BioSeq-Diabolo, while the biological sequence similarity scores calculated by BioSeq-Diabolo can also be used as the input features of BioSeq-BLM’s classification and sequence labelling models. We have added detailed description about their relationships in both the main text (introduction part) and the web site of Diabolo.

**C3.** Although figure 1 shows the similarities between sequence similarity analysis and semantics analysis, these similarities are not so clear. More explanations and discussions are needed, especially for their similarities among the individual steps shown in this figure.

**R3.** Good suggestion. While sentences determine their semantics of the corresponding natural language, biological sequences determine their structures and functions. The biological sequence similarity analysis is particularly similar with the semantics similarity analysis in NLP. Some tasks of protein structures and functions prediction are similar with the semantic matching tasks in NLP. We have added more descriptions of Figure 1 in its legend.

**C4.** In section 3.2, the concept of embedding is not clear. Does it mean extract the features of the biological sequences?

**R4.** Good point. the concept of embedding did mean the features of the biological sequences. Since the BioSeq-Diabolo integrated many deep learning networks, we use embedding to express the vectors or features of input deep learning networks.

**C5.** Learning to Rank is a method to combine different methods. This method seems to be better than the other unsurprised ones. As discussed in the corresponding section, it has been successfully applied to solve many problems in bioinformatics. The authors are suggested to show more details of Learning to Rank, because it is an important step in Diabolo.

**R5.** Good point. We have added detailed descriptions of the Learning to Rank in the Supplementary Materials.

**C6.** Written problems: the last column in Table 1, “Sequence similarities analysing” -> “Sequence similarity analysis”.

**R6.** Good point. We have corrected this written problem.

**C7.** The web server of Diabolo is a big advantage, and it is easy to use. I only need to feed the features, and the web server will output the desired analysis results. I validated it with both the examples provided by the authors and my own data, and I got the desired results. So I think the web server will popular with the other users as well, but Some improvements are desired. For term explanation, the authors are suggested to add the corresponding references of the terms, which will help the user to get the correct information. The main buttons (“Server”, “Tutorial” and “Document”) are suggested to be more clear and bigger.

**R7.** Constructive point. We have added the corresponding references of the terms and descriptions to help the user to get the correct information (<http://bliulab.net/BioSeq-Diabolo/doc/>), and adjusted the size of the main buttons.

**C8.** More discussions of the advantages and disadvantages of Diabolo are suggested to given in the conclusion part.

**R8.** Excellent suggestion. We have added more discussions of the advantages and disadvantages of BioSeq in the conclusion section.

**Reviewer #3**

**C.** In this work, by using the LTR integration method to analyze the similarity of different biological sequences (DNA, RNA, protein, disease, etc), a unified platform for systematically analyzing the similarity of homogeneous and heterogeneous biological sequences has been realized. The text is logically coherent, and the server functions provided are complete, powerful and useful. Here are some comments and suggestions to further enhance this study.

**R.** We very much appreciate Reviewer #3’s overall positive comments.

**C1**. In the three tests, the descriptions of the evaluation indicators are missing, and they don't appear in the supplementary material either.

**R1.** Good point. Exactly as suggested, we have added the descriptions of the evaluation indicators in the supplementary material.

**C2**. It is said that "The users only need to input the embeddings of the biological sequence data. BioSeq-Diabolo will intelligently identify the task, and then accurately analyse the biological sequence similarities based on biological language semantics." So how BioSeq-Diabolo intelligently identifies tasks is not explained in the paper.

**R2.** Good point. The biological sequence similarity analysis tasks can be divided into homogeneous biological sequence similarity analysis tasks and heterogeneous biological sequence similarity analysis tasks (see section 3.1). BioSeq-Diabolo will intelligently identify the specific tasks (homogeneous or heterogeneous biological sequence similarity analysis tasks) based on the input embeddings of the biological sequences and the parameters provided by the users. Please refer to section 3.3 for more details.

**C3**. In the protein function annotation task, the official evaluation metrics in CAFA are F-max and S_min, which are missing in this work.

**R3.** Very good suggestion. Exactly as suggested, these two evaluation indicators were provided. Please refer to **Table 4** and Supplementary Material.

**C4**. Lack of running time reports and performance evaluation of models under large-scale data. Estimation of time consumption is important for a user-oriented method.

**R4.** Good suggestion. The running time and performance evaluation of BioSeq-Diabolo under large-scale data were reported in **Fig. 6**. Please refer to section 4.1 for more details.

**C5**. Figure 1, a, the final protein should be "1D3B" instead of "D3B".

**R5.** Good point. We have corrected this mistake in **Figure 1**.

**C6**. Figure 4, "Learning to Rank" part, "Predicted diseases list for topic", "topic" is used in NLP background. Here it should be "Predicted diseases list for RNAs". The same problem exists in the Figure of the server (http://bliulab.net/BioSeq-Diabolo/server/).

**R6.** Good point. We have revised **Figure 4** and the Figure on the web server (http://bliulab.net/BioSeq-Diabolo/server/).

Finally, we would like to take this opportunity to thank you for taking time from your busy schedule to edit our manuscript. We are also very much indebted to the three anonymous reviewers, whose constructive comments are very helpful for strengthening the presentation of this paper.

With best regards,

Bin Liu, Ph.D

Professor
