# Supplementary material for: BioSeq-Diabolo: Biological sequence similarity analysis using Diabolo
Source: PLoS Comput Biol. 2023 Jun 20;19(6):e1011214. doi: 10.1371/journal.pcbi.1011214 (PMC10313010; doi:10.1371/journal.pcbi.1011214)
Supplement: Attachment — Submitted filename: Response Letter_v1.docx [file pcbi.1011214.s014.docx]

Dear Prof. Dr. Maxwell Wing Libbrecht,

Thanks for your e-mail and the attached review reports from three reviewers. In this revision, we have carefully revised all the points arising from the reviewers. Our point-to-point responses can be summarized as follows. For clarity, the reviewers’ comments are colored in green beginning with C (comment), while our responses in blue beginning with **R** (response).

**Reviewer #1**

**C.** It is revised well.

**R.** We very much appreciate Reviewer #1’s comments.

**Reviewer #2**

**C.** The authors have improved their work and addressed my comments. Happy to recommend this version for publication in PLoS CB.

**R.** We very much appreciate Reviewer #2’s comments.

**Reviewer #3**

**C.** The author has actively responded to our comments and resolved most of them. Here are some additional suggestions to further enhance this study.

**R.** We very much appreciate Reviewer #3’s suggestions. Our improvements to the study are as follows

**C1**. The author added "The details of the reported experiments" section in the supplementary material to further explain the details of the experiment. But as far as the protein function annotation problem is concerned, the experimental implementation has the following problem:

Generally speaking, when evaluating protein function annotation, most of the researchers will evaluate MFO, BPO, and CCO separately, instead of only evaluating CCO.

**R1.** Good point. Exactly as suggested, we have added the evaluation results of MFO and BPO in the supplementary material in **Table S4** and **Table S5**. It indicates that the predictors constructed by BioSeq-Diabolo are highly comparable with the state-of-the-art methods (same conclusion can be drawn from the evaluation results of CCO in **Table 4**).

**C2**. I think the training time should not be the focus of the evaluation. Instead, the experiment should focus on evaluating the time consumption between different approaches. That is, when the number of samples is the same, the time required for different methods to get the result from scratch.

**R2.** Good suggestion. Based on the same number of samples, the running time required for 9 different methods to get the result from scratch is show in the supplementary material in **Fig. S2**. With the same number of samples, interaction methods and representation methods (both are based on deep-learning) consume more time than distribution methods (based on traditional machine learning methods).

**C3**. "BioSeq- Diabolo will intelligently identify the specific tasks (homogeneous or heterogeneous biological sequence similarity analysis tasks) based on the input embeddings of the biological sequences and the parameters provided by the users." This section needs to specify how to identify specific tasks.

**R3.** Good point. The input embeddings of the biological sequences and the parameters for homogeneous and heterogeneous biological sequence similarity analysis tasks are different. For example, the input embeddings of protein sequences for protein remote homology detection (a homogeneous biological sequence similarity analysis task) need to have the same dimension and BioSeq-Diabolo will check the format of input embedding file before similarity analysis. BioSeq-Diabolo will remind users to input correctly formatted files and select necessary parameters to ensure the success of the analysis for specific tasks.

**C4**. In addition, users only need to input biological sequence data, but generating different input embeddings from biological sequence data requires different models. How does BioSeq-Diabolo select the corresponding model.

**R4.** Good point. BioSeq-Diabolo can’t generate embedding for biological sequence data. The task for embedding generation (feature extraction) can be implement by other biological sequence analysis tools, for instance, BioSeq-Analysis2.0 [1], BioSeq-BLM [2] and iLearnPlus [3]. In “BioSeq-Diabolo facilitates the protein remote homology detection” part (section 4.1), we use BioSeq-BLM to generate embedding for biological and then input the embedding to BioSeq-Diabolo. Different embeddings represent different features and property of biological sequences. More details about embedding generation can refer to BioSeq-BLM [2].

**C5**. The layout of Figure 4 is suitable, and many subplots are too small to be seen clearly. You can try to regroup the subplots. The theme of Diabolo does not need to be presented on this diagram, which hinders the normal layout of the diagram and can be reflected in your LOGO.

**R5.** Good suggestion. To display the schematic overview of BioSeq-Diabolo more clearly, we have regrouped the subplots and moved the flowchart to the supplementary materials (**Fig. S1**).

Finally, we would like to take this opportunity to thank you for taking time from your busy schedule to edit our manuscript. We are also very much indebted to the three anonymous reviewers, whose constructive comments are very helpful for strengthening the presentation of this paper.

With best regards,

Bin Liu, Ph.D

Professor

**Reference**

1.	Liu B, Gao X, Zhang H. BioSeq-Analysis2.0: an updated platform for analyzing DNA, RNA and protein sequences at sequence level and residue level based on machine learning approaches. Nucleic Acids Research. 2019;47(20):e127-e. doi: 10.1093/nar/gkz740.

2.	Li H-L, Pang Y-H, Liu B. BioSeq-BLM: a platform for analyzing DNA, RNA and protein sequences based on biological language models. Nucleic Acids Research. 2021;49(22):e129-e. doi: 10.1093/nar/gkab829.

3.	Chen Z, Zhao P, Li C, Li F, Xiang D, Chen Y-Z, et al. iLearnPlus: a comprehensive and automated machine-learning platform for nucleic acid and protein sequence analysis, prediction and visualization. Nucleic Acids Research. 2021;49(10):e60-e. doi: 10.1093/nar/gkab122.
